# Recent advances in understanding multiple sclerosis

**DOI:** 10.12688/f1000research.20906.1

**Published:** 2019-12-13

**Authors:** Peter K. Stys, Shigeki Tsutsui

**Affiliations:** 1 Department of Clinical Neurosciences, Hotchkiss Brain Institute, Medicine University of Calgary, Calgary, Alberta, Canada

**Keywords:** multiple sclerosis

## Abstract

Emerging data point to important contributions of both autoimmune inflammation and progressive degeneration in the pathophysiology of multiple sclerosis (MS). Unfortunately, after decades of intensive investigation, the fundamental cause remains unknown. A large body of research on the immunobiology of MS has resulted in a variety of anti-inflammatory therapies that are highly effective at reducing brain inflammation and clinical/radiological relapses. However, despite potent suppression of inflammation, benefit in the more important and disabling progressive phase is extremely limited; thus, progressive MS has emerged as the greatest challenge for the MS research and clinical communities. Data obtained over the years point to a complex interplay between environment (e.g., the near-absolute requirement of Epstein–Barr virus exposure), immunogenetics (strong associations with a large number of immune genes), and an ever more convincing role of an underlying degenerative process resulting in demyelination (in both white and grey matter regions), axonal and neuro-synaptic injury, and a persistent innate inflammatory response with a seemingly diminishing role of T cell–mediated autoimmunity as the disease progresses. Together, these observations point toward a primary degenerative process, one whose cause remains unknown but one that entrains a nearly ubiquitous secondary autoimmune response, as a likely sequence of events underpinning this disease. Here, we briefly review what is known about the potential pathophysiological mechanisms, focus on progressive MS, and discuss the two main hypotheses of MS pathogenesis that are the topic of vigorous debate in the field: whether primary autoimmunity or degeneration lies at the foundation. Unravelling this controversy will be critically important for developing effective new therapies for the most disabling later phases of this disease.

## Introduction

Multiple sclerosis (MS) is one of the most common causes of neurological disability in young adults. The etiology of MS has been intensively investigated for over a century, and although much has been discovered about the immunobiology, genetics, and epidemiology of this disease, the fundamental cause remains a mystery. MS is distinguishable from most other chronic neurological disorders by its unique fluctuating course; the majority of patients with MS present with early relapsing and remitting episodes of neurological and radiological worsening followed by varying degrees of recovery (relapsing-remitting MS, or RRMS) ^1^. In most patients, this initial relapsing remitting phase is followed years later by a more chronic progressive phase (secondary progressive MS, or SPMS), whereas a minority (≈15%) of patients begin with a progressive course from onset (primary progressive MS, or PPMS) for unknown reasons.

Traditionally, the etiology of MS was based on an “outside-in” autoimmune hypothesis whereby dysregulated auto-reactive T cells in the periphery cross into the central nervous system (CNS) parenchyma and, together with macrophages and B cells, proceed to attack various CNS elements, where myelin is a prominent target. These inflammatory events, resulting in a relapsing-remitting clinical course, likely contribute independently to accumulating CNS injury. As a result, most work in the field has been directed at the autoimmune inflammatory nature of the disease, resulting in over a dozen US Food and Drug Administration/European Medicines Agency–approved treatments to date ^2^. This traditional model is being challenged by a competing theory proposing that the initial malfunction occurs *within* the CNS, as suspected for other neurodegenerative disorders such as Alzheimer’s and Parkinson’s diseases ^3– 7^. This alternative “inside-out” hypothesis argues that MS is a primary degenerative disease and is accompanied by varying degrees of inflammation, leading to the release of antigenic cell components such as myelin oligodendrocyte glycoprotein, myelin basic protein, and proteolipid protein ^8, 9^. This chronic shedding of auto-antigens secondarily promotes an autoimmune inflammatory response in the predisposed host, in turn driving additional degeneration in a vicious cycle. The extent of inflammatory activity, which varies by patient and over time within the same individual, determines the spectrum of MS phenotypes. Importantly, the “inside-out” hypothesis maintains that primary degeneration is present from the start (probably years before the first overt clinical symptoms) and continues throughout the entire course of the disease. Given that CNS inflammation in MS can be well controlled with modern therapies, the current challenge is progressive disease, during which most irreversible disability occurs ^10, 11^. At present, we neither understand the mechanisms driving this phase of MS nor have very effective treatment options for patients in whom degeneration predominates in the absence of overt inflammation. The goal of this commentary is to touch on some recent findings and discuss recent advances regarding progressive MS pathogenesis.

## MS stages and clinical progression

Much effort has been expended in an attempt to accurately classify the various stages of MS. Clinical relapses at a younger age, coupled with radiological evidence of CNS inflammation, provide good support for the earlier relapsing-remitting phase of MS. This may be important from a practical point of view to guide clinical therapeutic decisions given the many options for effective anti-inflammatory agents that are currently available. But with time, identifying the transition from relapsing to secondary progressive disease often becomes problematic; absence of clinical relapses does not imply absence of inflammatory activity on magnetic resonance imaging, and absence of inflammatory radiological lesions does not imply lack of inflammation at the tissue level. Indeed, it has been proposed that MS is a condition in which disability progression is driven in two stages, and early relapsing inflammatory activity has little influence on the second, later stage of progressive deterioration ^12^. Therefore, identifying the transition to secondary progression has important practical implications because it appears that our current anti-inflammatory drugs offer little benefit in the absence of ongoing inflammatory activity; continuing treatment entails expense and potential risk from potent immunosuppressants with potentially little benefit in return. This is not to say that inflammation disappears at later stages. Indeed, recent studies show that substantial inflammatory lesion activity persists well into the progressive phase and, perhaps counterintuitively, that SPMS and PPMS samples exhibit a higher lesion load compared with relapsing subjects ^13^. Moreover, there are few differences in lesion morphology or immunopathology in SPMS versus PPMS brain ^14^, even though decades of recurrent inflammatory attack preceded the former. Instead, the quality of ongoing inflammation seems to shift in favor of activated macrophages/microglia. The MS brain in progressive stages shows ongoing cortical demyelination and slowly expanding demyelinating lesions in the white matter with such lesions recently shown to be important predictors of future disability ^15^. Interestingly, there is scant lymphocytic activity (B cells are a peculiar exception; see below); instead, microglial activation is seen at the borders of expanding lesions ^16^. This suggests that adaptive autoimmunity plays a lesser role at this stage and that innate responses predominate. A key question is whether innate immune cells are somehow dysregulated and become primary drivers of degeneration, or simply react as they are expected to, against an unidentified primary injury that itself is the underlying cause of tissue destruction. Taken together, recent data seem to support the idea that all types of MS might be variations of a similar underlying pathophysiological theme and that degeneration and concomitant inflammation begin early and proceed inexorably, regardless of whether relapse activity is a prominent feature ^17^. If so, why some patients assume a more relapsing inflammatory pattern at first whereas others begin later with a progressive course is unclear. Here, the genetic background and environment likely play major roles in programming the various clinical phenotypes without the need to invoke fundamentally different disease mechanisms.

## The genetics of MS

In MS, as in most other neurodegenerative diseases, genetic influences play an important role as well. However, unlike in a number of other neurodegenerative disorders, where specific mutations have been identified as causes for at least a subset of these, such mutations have yet to be identified in MS ^18– 20^. Instead, a large number of susceptibility loci, the vast majority of which are related to immune function, have been found through genome-wide association studies (GWASs) ^20– 27^. Together, these studies unequivocally confirm the importance of immune-related genes, and genetic maps from a very recent GWAS further implicate both the adaptive and innate immune systems ^28^. Examples include the human leukocyte antigen (HLA) class II (DP, DQ, and DR) haplotypes which demonstrate an association with MS ^29^, although contradictory results were shown in various studies among different races ^30, 31^. The HLA-DRB1*15:01 allele is one of the most intensively studied ^31, 32^, exhibiting a consistent association with a lower age of onset, greater white matter lesion volume, and faster brain atrophy in RRMS ^33^. In addition, a recent report suggested that the high-risk HLA genotype (two predisposing haplotypes) associates with significant reduction in whole brain and grey matter volumes compared with medium- or low-risk genotypes (one or no predisposing haplotypes) ^34^. Many loci outside the major histocompatibility complex (MHC) have also been correlated with MS risk and involve other immune pathways, including B-cell activation, cytokine release, and activation of immune cells both in the periphery and within the CNS ^27^. This study identified additional novel candidates associating with regulators of CD4 ^+^ Th1 and Th17 induction and apoptosis ^27^. Genes involved in neurodegeneration, such as mitochondrial genes *CRYAB*, *CB1* and *Prnp*, have been studied, but associations with PPMS risk seem to involve mainly variants of immune-related genes, similar to what was found with relapse-onset MS ^32^. However, none of these has a strong association with progressive MS, suggesting that these immune-related factors are unlikely to be causative but instead might determine the intensity of autoimmune reactivity to a degenerating brain. Genetic studies on patients with PPMS are less comprehensive because of the much lower incidence of this MS phenotype. However, despite the striking difference in clinical phenotypes between relapse-onset MS and PPMS, clear genetic differences have not emerged ^32^. Interestingly, however, MS patients exhibiting a higher degree of neurodegeneration had variants in genes related to glutamate signaling ^35, 36^, supporting the concept of a neurodegenerative underlay driven at least in part by “chronic excitotoxicity”. Moreover, such chronic excitotoxicity is likely further exacerbated by glutamate released by immune cells ^37^. Finally, we recently showed that primary myelin damage is a strong trigger for a secondary immune reaction driven in large part by biochemically altered myelin proteins ^38^; it is quite likely that chronic excitotoxicity, originating from both the as-yet-unknown underlying degenerative process(es) and glutamate release from infiltrating immune cells, promotes a persistent source of pathological myelin products resulting in a vicious cycle of degeneration–inflammation–more degeneration ^39^. Taken together, data so far unequivocally support the contribution of multiple immune-related genetic loci to relapsing-remitting inflammatory MS, but a direct causal relationship remains elusive. A thought-provoking parallel can be drawn with genetic studies of human susceptibility to tuberculosis (TB), for instance. The genetics of TB, like those of MS, are complex, but there are interesting similarities, including the greater susceptibility of mono- versus dizygotic twins and association with HLA class I and II ^40^. Because we now know the underlying cause of TB, these genetic studies instead provide important insight into host susceptibility and response to an external pathogen. One could similarly argue that studies of MS genetics also provide information on host susceptibility and response to some causative factor that nonetheless remains unknown. By extension, elucidating multiple immune-related genetic loci associated with MS may not bring us any closer to identifying the root cause any more than such studies would have helped in isolating the TB bacillus.

## Environmental factors

In addition to immunogenetics, MS risk is strongly associated with environmental factors, in particular infection with Epstein–Barr virus (EBV), sun exposure/vitamin D deficiency, and smoking ^41, 42^. Past EBV exposure as evidenced by seropositivity to the virus is virtually a prerequisite for developing MS ^43, 44^. However, given the very high global prevalence of EBV exposure, clearly the converse is not true; that is, only a small minority of exposed individuals will develop MS, indicating that EBV may be necessary but not sufficient to trigger the disease. Epidemiological evidence supporting the important role of this virus includes observations that patients with a history of symptomatic EBV infection carry a higher risk of developing MS ^45^, and the risk of MS dramatically increases in seronegative individuals after seroconversion ^46^. These strong associations lead to the hypothesis that B-lymphotropic EBV infection of CNS-infiltrating B cells may somehow drive MS pathology ^47^, although such a direct causative role of EBV remains controversial as some groups report absence of EBV infection in MS brain ^48, 49^. A possible explanation for this discrepancy is that EBV is one of many possible triggers of secondary autoimmune response or that EBV alone is not sufficient ^50^. Although there is no consensus regarding a direct role of EBV-infected B cells as a primary cause of MS, increasing evidence, including therapeutic strategies, indicates a potentially important role in MS pathogenesis. For instance, recently, a preliminary clinical trial of autologous EBV-specific T-cell therapy showed clinical improvement in patients with progressive MS ^51^. Although only 10 subjects underwent treatment, this small study provides some of the most direct evidence to date of a potentially central role of EBV-infected B cells in MS. Moreover, the fact that patients in the progressive phase, a phase largely resistant to current therapies, experienced improvement is equally noteworthy. Whether the beneficial effects of T cell–mediated killing of EBV-infected B cells were due to a reduction of disease-causing immunoglobulin production, or of other toxic soluble factors produced by B cells ^52^, remains to be seen.

## MS Pathology

Although an inflammation-mediated demyelination of white matter tracts with partial preservation of axons is a hallmark of MS, recent advances in histopathological/imaging techniques have emphasized the prevalence of cortical demyelination, where lymphocyte or macrophage infiltrates are limited ^53– 57^. Cortical demyelination and grey matter demyelination in cerebellar cortex, hippocampus, and deep grey matter nuclei are major histological features of progressive MS ^58– 61^. Evidence suggesting that grey matter involvement is related to disease activity and more aggressive forms is growing. Recent studies have shown that cortical and deep grey matter lesions in the thalamus, caudate, putamen, and cerebellar cortex during the early stage of disease are independent of white matter pathology ^62, 63^. These recent findings strongly support the hypothesis proposed a decade ago that neurodegeneration becomes independent of inflammation during progressive MS ^4^ and that MS could be a primary degenerative disease. In support of these findings, inflammatory relapses were recently reported to be associated with transient short-term disability, but long-term worsening was largely independent of relapses ^17^. The authors proposed the concept of “silent progression” that is consistent with an underlying degenerative process, which proceeds largely independently of autoimmune inflammation ^17^. The age and gender differences between relapse-onset MS (younger age at onset and greater prevalence in women) and PPMS (later age at onset and equal prevalence among the sexes) do not necessarily imply a fundamentally different etiology. Just as with TB susceptibility discussed above, such differences can be readily explained by the known difference in autoimmune predilection which is greater in women ^64^; a subject afflicted with the same underlying cytodegeneration, but more prone to react with an inflammatory lesion, would naturally present earlier (inflammatory lesions in eloquent regions of the CNS will result in more obvious clinical disability). As age and accompanying immune senescence progress, MS at later ages would be expected to exhibit less inflammation.

In contrast to RRMS, which has major involvement of peripheral immune cell infiltration in actively demyelinating plaques, progressive MS may involve the development of compartmentalized pathological processes within the resident CNS cells. Microglia are the most abundant resident macrophage-like cells in the CNS and constitute one of the key cell types that could trigger neurotoxic pathways leading to progressive neurodegeneration or, in contrast, could exert important roles in promoting neuroprotection, downregulation of inflammation, and stimulation of repair. Activated microglia exhibit cytoplasmic hypertrophy, retraction of processes, and upregulation of MHC class II molecules and pro-inflammatory cytokines ^65^. Microglial activation not only is observed in early active lesions but also accompanies diffuse axonal injury in normal-appearing white and grey matter of progressive MS lesions ^66^. In addition, microglial nodules (a clustering of activated microglia) are abundant in the areas adjacent to plaques, particularly in patients with progressive MS ^67^. In an attempt to understand the neuropathological processes occurring in progressive MS, a positron emission tomography (PET) imaging method with radioligands binding to the 18-kDa translocator protein (TSPO) molecule on activated microglia was developed for monitoring these cells in living patients with MS ^68^. This revealed that TSPO binding was significantly increased in the normal-appearing white matter (NAWM) of patients with SPMS ^69– 71^ but only modestly in the NAWM of RRMS brain ^70^. In PPMS, such studies are still limited ^72^. Most recently, TSPO-PET studies showed significantly increased signals in subcortical grey matter regions, especially in thalamus in SPMS, and this was associated with accelerated brain atrophy ^73^. Thus, activated microglia are observed in progressive MS; however, what is not yet known is whether they contribute to ongoing degeneration or reflect a normal reaction to injury and serve a protective role.

## Mitochondrial dysfunction in MS

Mitochondria play a central role in the supply of energy to electrically active axons and neurons. Dysfunction of this organelle likely contributes to “virtual hypoxia” ^74^ and may be especially relevant for chronically demyelinated axons where an action potential may exact a higher toll on energy supply. This imbalance of supply versus demand could further exacerbate degeneration of vital tracts through energy failure, induction of apoptosis, and enhanced production of reactive oxygen species ^75^. Therefore, this topic has been gaining interest as an important mechanism of neuronal death. Oxidative burst in activated microglia produces reactive oxygen and nitrogen species which contribute to mitochondrial dysfunction ^76^. Therefore, the activated microgliosis described above could exacerbate axo-glial injury in white matter that does not exhibit overt pathological changes (NAWM). A marker of mitochondrial dysfunction, cerebrospinal fluid (CSF) lactate showed a positive correlation with disease onset, severity, and progression ^77– 79^, suggesting that this organelle might be under stress from the earliest stages of the disease. A recent study reported that ceramides in CSF from patients with progressive MS induced mitochondrial elongation and reduced respiratory chain complex activity in cultured neurons, providing evidence of a specific factor contributing to mitochondrial injury ^80^. This supports previous findings that decreased functional activity of mitochondrial respiratory chain complexes in progressive MS motor cortex neurons may contribute to accumulating neurological disability in patients with MS ^81^. In addition, respiratory chain complex IV–deficient neurons and choroid plexus (CP) epithelial cells are more abundant in MS brain and this respiratory enzyme deficiency is caused by a high level of mitochondrial DNA (mtDNA) deletions ^82, 83^ (reviewed in 84). Interestingly, no significant change in the extent of respiratory-deficient skeletal muscle fibers was found in patients with progressive MS compared with age-matched controls ^85^, indicating that such mitochondrial changes are induced within the CNS. The cause of this mitochondrial damage, which likely plays an important role in further compromising the health and function of brain neurons and axons, is not completely understood.

## Conclusions

It is very clear that both degenerative and immune processes are involved in the pathophysiology of MS. Unfortunately for the MS researcher, the closely intertwined relationship between these two processes, continuing throughout all phases of the disease, makes it extremely difficult to definitively resolve the “outside-in” versus “inside-out” controversy. In our opinion, emerging evidence, as summarized in this commentary, is providing increasing support in favor of a primary degenerative etiology. However, as with most other neurodegenerative disorders, the proximal trigger is unknown. Regardless, what is clear is that understanding the mechanisms of chronic progression in MS is currently the major challenge because this is the phase that contributes most to irreversible disability. The lack of insight into mechanisms of progression is largely responsible for the extremely limited treatment options currently available to patients with progressive MS. A shift in thinking about this disease, with greater consideration given to a potential underlying degenerative etiology, will spur new and original research directions that will eventually unravel the mystery and provide more effective therapeutics to mitigate the disabling progressive phase of MS.
